# Ketamine, Esketamine, and Arketamine: Their Mechanisms of Action and Applications in the Treatment of Depression and Alleviation of Depressive Symptoms

**DOI:** 10.3390/biomedicines12102283

**Published:** 2024-10-09

**Authors:** Piotr Kawczak, Igor Feszak, Tomasz Bączek

**Affiliations:** 1 Department of Pharmaceutical Chemistry, Faculty of Pharmacy, Medical University of Gdańsk, 80-416 Gdańsk, Poland; tomasz.baczek@gumed.edu.pl; 2 Institute of Health Sciences, Pomeranian University in Słupsk, 76-200 Słupsk, Poland; igorfeszak@gmail.com; 3 Department of Nursing and Medical Rescue, Institute of Health Sciences, Pomeranian University in Słupsk, 76-200 Słupsk, Poland

**Keywords:** ketamine, esketamine, arketamine, depression, psychiatric treatments

## Abstract

Research over the past years has compared the enantiomers (S)-ketamine (esketamine) and (R)-ketamine (arketamine) of the previously known racemic mixture called ketamine (R/S-ketamine). Esketamine has been found to be more potent, offering three times stronger analgesic effects and 1.5 times greater anesthetic efficacy than arketamine. It provides smoother anesthesia with fewer side effects and is widely used in clinical settings due to its neuroprotective, bronchodilatory, and antiepileptic properties. Approved by the FDA and EMA in 2019, esketamine is currently used alongside SSRIs or SNRIs for treatment-resistant depression (TRD). On the other hand, arketamine has shown potential for treating neurological disorders such as Alzheimer’s, Parkinson’s, and multiple sclerosis, offering possible antidepressant effects and anti-inflammatory benefits. While esketamine is already in clinical use, arketamine’s future depends on further research to address its safety, efficacy, and optimal dosing. Both enantiomers hold significant clinical value, with esketamine excelling in anesthesia, and arketamine showing promise in neurological and psychiatric treatments.

## 1. Introduction

In the 1950s, Parke-Davis researched cyclohexylamines to discover an effective induction agent with analgesic properties, identifying CI395 and CI400. In 1965, Guenter Corssen and Edward Domino tested CI-581, or ketamine, which proved effective for pediatric patients, though further adult trials were needed. By 1968, ketamine was established as a safe anesthetic and analgesic that preserved the airway’s reflexes without significant post-operative nausea or hypotension. However, it was associated with vivid dreams and hallucinations during recovery, particularly in adults, which led to its preference in pediatric care. In the early 1970s, ketamine was introduced in the UK but was deemed unsuitable as a sole anesthetic for adults, although it remained popular for pain relief and in veterinary medicine [1,2,3,4,5,6].

Ketamine’s dissociative anesthetic properties have led to its unique role in clinical practice, despite early drawbacks such as emergence reactions and cardiovascular stimulation, which limited its use as a standalone anesthetic [7,8,9,10,11]. Combining ketamine with benzodiazepines helped reduce these side effects [12,13,14,15]. While ketamine is often used as a racemic mixture (R/S-ketamine), the more potent (S)-enantiomer has been preferred for its better anesthetic and analgesic outcomes, though it can still cause somatic and psychotomimetic effects, including perceptual disturbances and dissociation [16,17].

Ketamine metabolites, particularly hydroxynorketamine (HNK), have shown antidepressant properties in preclinical studies, suggesting potential treatments that retain therapeutic benefits while reducing side effects [18,19,20,21,22]. (S)-ketamine has been found to cause fewer psychotomimetic effects compared with the racemic mixture, while providing stronger hypnotic and analgesic effects, faster recovery, and fewer cognitive side effects, making it more suitable for anesthesia [23,24,25]. Both (S)- and (R)-ketamine affect multiple systems, with (S)-ketamine having a higher affinity for the sigma receptors, contributing to psychotomimetic effects. (R)-ketamine, however, may provide more lasting antidepressant effects by promoting synaptogenesis, as evidenced by animal studies and early clinical trials in treatment-resistant depression (TRD) [23,26,27].

Over 40 years of research comparing (S)- and (R)-ketamine has highlighted the superior pharmacological profile of (S)-ketamine, which is about three times more potent as an analgesic and 1.5 times stronger as an anesthetic compared with (R)-ketamine. Human studies have confirmed these findings, with (S)-ketamine offering better anesthesia quality and fewer side effects, such as involuntary movements, compared with the racemic mixture [28,29]. Both (S)-ketamine and (R)-ketamine show antidepressant effects through different mechanisms. (S)-ketamine blocks N-methyl-D-aspartate (NMDA) channels and activates the opioid receptors, while (R)-ketamine likely acts on the sigma-1 receptors. Both enantiomers inhibit glycogen synthase kinase-3 beta (GSK3β), contributing to their antidepressant effects. In major depressive disorder (MDD) patients, (S)-ketamine nasal spray has antidepressant effects but is less effective than intravenous racemic ketamine in terms of its response rate, duration, and antisuicidal effects [30,31,32].

(S)-ketamine has been used for over 30 years in clinical settings for pain relief and anesthesia, primarily by blocking NMDA receptors and interacting with opioid, monoamine, adenosine, and purinergic receptors. It also affects α-amino-3-hydroxy-5-methyl-4-isoxazolepropionic acid (AMPA) receptors, metabotropic glutamate (mGlu) receptors, and L-type calcium channels. Its activation of the sympathetic nervous system makes it suitable for anesthesia and sedation in unstable patients. Its neuroprotective, bronchodilatory, antihyperalgesic, and antiepileptic properties have expanded its use to emergency settings since the 1990s. It is particularly beneficial for neurological injuries, bronchospasm, seizures, and sepsis, with fewer psychotropic side effects compared with racemic ketamine [33].

(R)-ketamine has shown positive results in animal models of neurological disorders, with potential for treating social cognitive deficits by restoring anterior insular cortex (aIC) function [34]. Lipopolysaccharide (LPS), a bacterial endotoxin, may play a role in neurological disorders such as Alzheimer’s disease (AD), other dementias, and Parkinson’s disease (PD) [35]. Studies indicate that (R)-ketamine may reduce systemic inflammation, splenomegaly, and behavioral problems in mice treated with LPS [36], as well as in models of depression, colitis, and sepsis [37,38]. However, its mechanisms of action remain unclear, requiring further research to explore its molecular pathways and identify new therapeutic targets [39,40,41,42,43,44]. Depression is a risk factor for neurological conditions such as AD, PD, and stroke, with a strong connection between psychiatric and neurological disorders [45,46]. A Phase II study by Perception Neuroscience on (R)-ketamine (PCN-101) in treatment-resistant MDD patients suggested that it may help prevent demyelination and aid in remyelination in multiple sclerosis (MS) patients [47,48]. (R)-ketamine may also help manage depression in neurological disorders such as dementia, PD, MS, and stroke, potentially serving as a treatment or preventive drug. However, further randomized clinical trials are necessary to confirm its efficacy in delaying or preventing these conditions. Overall, (R)-ketamine has shown strong antidepressant and anti-inflammatory effects, but further research is needed to understand its molecular mechanisms [47].

This review underscores the therapeutic promise of R/S-ketamine, specifically (S)-ketamine and (R)-ketamine, by examining their mechanisms and their application in addressing depression and related conditions within modern therapeutic frameworks. This article provides an in-depth evaluation of both (S)-ketamine and (R)-ketamine as viable antidepressant options, outlining several strengths as well as areas that warrant additional exploration. It recognizes that while (S)-ketamine has demonstrated rapid antidepressant effects in controlled clinical trials, its effectiveness in real-world settings remains questionable, particularly among larger and more diverse patient populations, where the results have not consistently outperformed those of a placebo when used alongside traditional oral antidepressants. This suggests that its effectiveness may be limited in practical applications. The safety issues related to (S)-ketamine usage are significant, as it can result in side effects such as dissociation, dizziness, and cardiovascular complications, potentially hindering long-term treatment adherence. Additionally, concerns about the risk of misuse and dependence further restrict its application in nonclinical environments. This article stresses the necessity for more research on (S)-ketamine, especially concerning its long-term effectiveness, safety profile, and mechanisms of action, including its interactions with various neurotransmitter systems. It proposes that personalized medicine approaches and improved patient selection criteria could optimize treatment outcomes, particularly for individuals with psychiatric comorbidities. In relation to (R)-ketamine, this review points out a deficiency in the comprehensive clinical data, with the majority of evidence coming from preclinical studies. It advocates for large-scale randomized controlled trials to determine the antidepressant efficacy of the drug and establish optimal dosing strategies, highlighting the variability in the biological pathways linked to its effects. By tackling these limitations and suggestions, this review establishes a solid foundation for future research into the therapeutic potential of ketamine in treating depression.

The literature in this article was accessed through PubMed using this review article’s keywords: ketamine, esketamine, arketamine, depression, and psychiatric treatment (with a direct link provided in the Supplementary File, leading to the relevant sources used for extraction of the information). This search produced around 610 results, from which over 130 articles were selected, focusing on the most recent reports published on or after 2019, along with some older studies required to explain the theoretical foundations.

## 2. Ketamine

**Figure 1:**
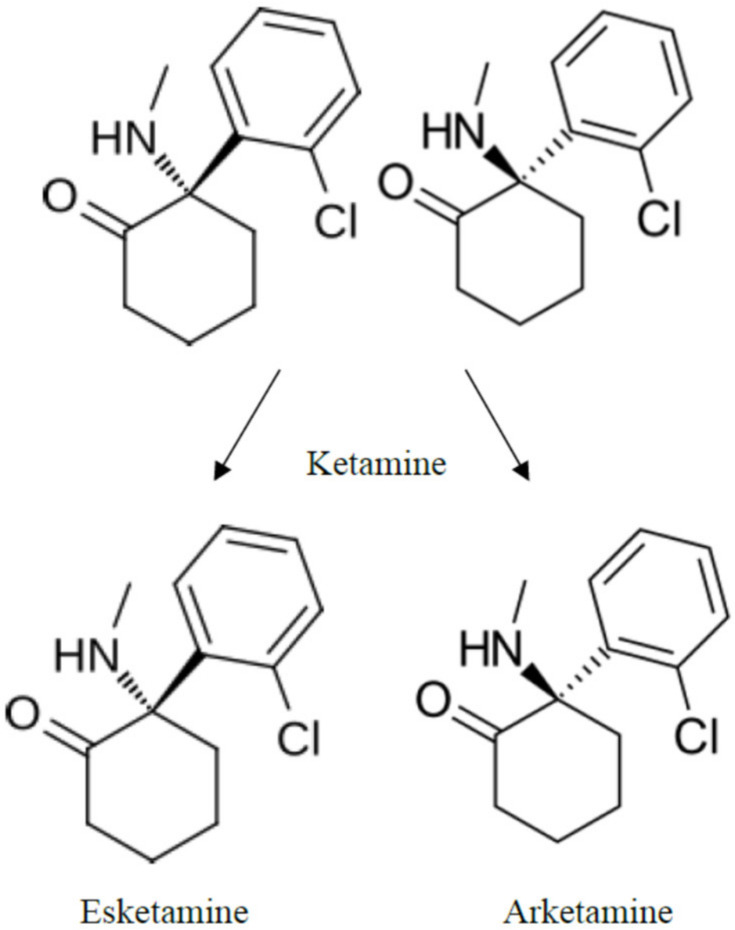
Structural formulas of R/S-ketamine, S-ketamine, and R-ketamine.

Ketamine or R/S-ketamine is a combination of two enantiomers, S-ketamine and R-ketamine (Figure 1).

Ketamine, an NMDAR antagonist traditionally used as an anesthetic, has gained attention as a novel treatment for depression, particularly for severe and treatment-resistant cases. Meta-analyses have offered important insights into the factors that may influence its effectiveness, such as a better response in unipolar depression compared with bipolar depression, prolonged benefits with repeated treatments, different methods of administration, and indirect comparisons between the efficacy of racemic ketamine and the esketamine enantiomer [49,50]. MDD is a debilitating condition affecting millions globally, contributing significantly to health and socioeconomic burdens. According to the World Health Organization (WHO), depression ranks third in terms of its global disease burden, placing an immense strain on the societal costs due to disability. Traditional antidepressants that target the monoamine system take 4–12 weeks to show improvements. However, recent studies have highlighted the role of glutamate, particularly NMDARs, in depression. Glutamate, the main excitatory neurotransmitter, plays a key role in neurodevelopment, memory, learning, and nerve growth. Ketamine, an NMDAR antagonist, has shown rapid antidepressant effects at low doses. A single dose can quickly alleviate depressive symptoms, with effects lasting up to a week, suggesting its influence on neuroplasticity. Multiple studies have reported significant symptom remission within a week of ketamine administration, and its efficacy has been demonstrated from Day 1 in patients with both unipolar and bipolar depression. In addition to reducing depressive symptoms, subanesthetic doses of ketamine have been effective in managing suicidal ideation [51].

Given the limitations of current antidepressants and the insufficient evidence supporting the monoamine deficiency theory of depression, there is growing interest in exploring new targets for antidepressant treatment, particularly within the glutamatergic system. Ketamine hydrochloride, a noncompetitive and nonsubtype-selective NMDAR antagonist, has primarily been used as an anesthetic since the 1960s at doses of 1–3 mg/kg. A groundbreaking pilot study in the year 2000 revealed ketamine’s rapid and robust antidepressant effects when administered intravenously at subanesthetic doses to individuals with TRD. Subsequent randomized clinical trials and meta-analyses have reinforced ketamine’s potential as a novel and effective antidepressant. Most studies administered a single 40-min IV infusion of (R,S)-ketamine (racemic mixture) at 0.5 mg/kg, showing response rates of 50–70% in TRD patients. Many individuals reported significant symptom relief, including reductions in depressed mood, anhedonia, and suicidal thoughts, within 2 h of administration, with effects peaking at 24 h and lasting up to 2 weeks. Ketamine’s rapid antidepressant effects contrast sharply with the delayed onset of traditional antidepressants and challenge the monoamine deficiency hypothesis of depression. While monoaminergic systems may not be the primary pathway for mood regulation, they might influence downstream signaling pathways targeted by ketamine. The prolonged antidepressant response observed after a single ketamine infusion, despite its short plasma half-life of 2.5 h, suggests that ketamine’s effects are mediated by activating key downstream signaling pathways rather than its direct receptor interactions. Despite its promising results, ketamine administration, even at subanesthetic doses, can cause mild and temporary dissociative effects, neurocognitive and sensorimotor disturbances, and transient increases in heart rate and blood pressure. Additionally, as ketamine is sometimes abused recreationally, there is concern about potential neurotoxic effects from prolonged use. Identifying the precise mechanisms behind ketamine’s antidepressant effects could lead to the development of new rapid-acting antidepressants with fewer side effects and a broader clinical application [52].

Ketamine is an open-channel blocker of ionotropic NMDARs, which has been recognized for its rapid antidepressant effects in individuals with depression and treatment-resistant depression. This finding has not only led to the development of new, effective treatments for mood disorders but has also offered valuable insights into the neurobiology of these conditions. Additionally, it has revealed key mechanisms of synaptic plasticity that are crucial for its therapeutic impact. The discovery of ketamine’s rapid antidepressant effects in patients with depression and treatment-resistant depression has sparked a revival in both clinical and preclinical neuropsychiatry. Ketamine’s swift efficacy suggests that symptoms of depression can be quickly alleviated, even in patients with long-term treatment challenges. Preclinical studies have tested this idea, finding that retinoic acid receptor activation can induce rapid homeostatic plasticity similar to ketamine, though it does not involve NMDARs or their signaling pathways. While retinoic acid signaling is not required for ketamine’s antidepressant effects, its direct activation can produce similar rapid antidepressant-like results, indicating that targeting homeostatic plasticity could be sufficient for antidepressant action. However, this hypothesis needs clinical validation. Another important area of research is maintaining ketamine’s antidepressant effects. One strategy could be to use ketamine to achieve rapid symptom relief and then target specific downstream signaling pathways to extend its effects, potentially reducing the need for repeated ketamine dosing. This approach could help mitigate the need for ongoing ketamine treatment in long-term depression management. Ketamine’s action has shifted the research focus from traditional “slow” neurotransmission, involving monoaminergic systems, to the role of fast glutamatergic neurotransmission in mood disorders. To fully leverage the potential of fast neurotransmission in neurotherapies, it is crucial to develop new therapeutics that target these rapid signaling mechanisms without disrupting their essential functions in sensory processing, learning, and memory. Investigating parallel signaling pathways and multiple mechanisms within single synapses could lead to new treatments for neuropsychiatric disorders, aiming towards fewer side effects, rapid onset, and sustained efficacy. Overall, ketamine’s ability to induce homeostatic plasticity rather than addressing the underlying causes of depression suggests it may provide a temporary alleviation of symptoms. Identifying compounds that specifically target homeostatic plasticity could represent a promising new therapeutic strategy [53].

**Figure 2:**
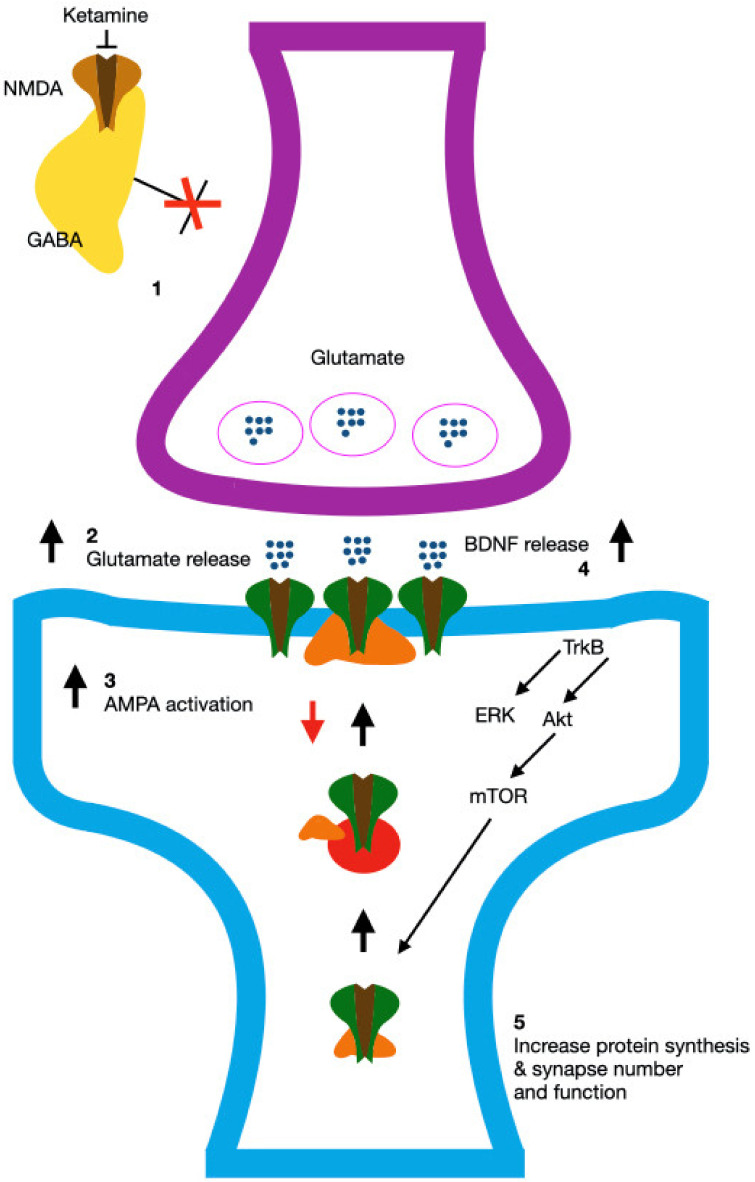
The proposed mechanism of ketamine’s antidepressant effect, according to [54], involves the suppression of tonic GABAergic activity (1), which leads to a surge in glutamate release and metabolism (2); this increased glutamate activity, through AMPA receptors (whose surface expression may be boosted by the reduced spontaneous activity of NMDA receptors) (3), promotes BDNF-dependent (4) synaptic growth (5), ultimately contributing to rapid and sustained antidepressant effects. Akt, protein kinase B; ERK, extracellular signal-regulated kinase; mTOR, mammalian/mechanistic target of rapamycin; TrkB, tropomyosin kinase B.

The suggested mechanism through which ketamine exerts its antidepressant effect is illustrated in Figure 2, while Table 1 shows a comparison of the antidepressant and side effects of racemic ketamine, esketamine, and arketamine in both humans and animals.

## 3. Esketamine

(S)-(+)-ketamine or (S)-ketamine, also known as esketamine, was approved by the Food and Drug Administration (FDA) and European Medicines Agency (EMA) in 2019, and is the only glutamatergic neuromodulatory agent authorized to augment the effects of selective serotonin reuptake inhibitors (SSRIs) or serotonin–norepinephrine reuptake inhibitors (SNRIs). Due to the high rates of partial responses or nonresponse to existing antidepressants, researchers are exploring new pharmacological agents that target mechanisms beyond monoaminergic neurotransmission. While numerous compounds have undergone Phase II and III clinical trials, it remains challenging to predict which will enter the market in the coming decades. So far, only esketamine and brexanolone—a positive allosteric modulator of the gamma-aminobutyric acid A (GABA-A) receptor—have been FDA-approved for supervised use in patients with treatment-resistant depression and post-partum depression, respectively. Furthermore, tolerability issues with the current antidepressants underscore the need for novel pharmacological options to treat major depression [55]. Esketamine nasal spray is recommended for adults with MDD who have not responded to at least two antidepressants and are currently experiencing a moderate or severe depressive episode. Both the FDA and EMA have outlined strict monitoring protocols for esketamine’s use, including assessments before and after administration. Esketamine works by blocking NMDARs, which are glutamate receptors. This leads to increased glutamate release, activating other receptors that enhance synaptogenesis and improve signaling via neurotrophic factors in brain regions involved in mood regulation. It also restores dopamine transmission, which helps reduce symptoms such as anhedonia (loss of pleasure), though it may cause psychotic-like effects due to dopamine release in certain brain areas. Esketamine’s fast action is linked to the stimulation of the mammalian/mechanistic target of rapamycin complex 1 (mTORC1) signaling pathway, which supports synapse formation and brain-derived neurotrophic factor (BDNF) production. Recent research has highlighted the role of glutamatergic mechanisms in depression, with abnormal glutamate levels observed in individuals with mood disorders. Ketamine, related to esketamine, is thought to work by blocking NMDARs on GABA neurons, disinhibiting pyramidal neurons, and enhancing synaptic plasticity via mTORC1 signaling. This mechanism leads to increased synapse formation in the prefrontal cortex, offering rapid antidepressant effects. However, other drugs targeting NMDARs, such as memantine and lanicemine, have not shown similar efficacy in treating depression, suggesting that additional mechanisms may be involved [31]. Intravenous esketamine has shown rapid and lasting effects in patients with MDD who do not respond to standard treatments. It has also demonstrated positive outcomes in treatment-resistant patients at an immediate risk of suicide, as seen in Phase II studies. Similarly, intranasal esketamine has been investigated for its rapid antidepressant effects in patients with depression and suicidal thoughts, with notable benefits observed after just one dose. Esketamine’s antisuicidal effects are a major reason for its study, as traditional antidepressants struggle to manage suicidal behavior in patients with MDD. Recent advances in combining genomic and clinical evaluations to identify markers of suicide risk have sparked interest in drugs that affect neural connectivity, immune responses, and inflammation. Dysregulated glutamate neurotransmission is thought to play a key role in suicidal behavior, making ketamine and esketamine promising treatments. Along with blocking NMDARs, ketamine also affects the opioid, serotonin, muscarinic, and nicotinic receptors, which may contribute to its antisuicidal properties. However, some studies have cautioned that the enthusiasm for esketamine as a treatment for suicidal patients should be reassessed based on real-world experience. It is crucial to combine careful suicide risk assessments with a compassionate understanding of the patients’ subjective experiences. Suicide, often driven by overwhelming negative emotions or acute distress, should be viewed as distinct from typical symptoms of depression, requiring a nuanced approach to treatment. The metabolite (S)-norketamine, formed through the metabolism of esketamine by cytochrome P450, has been found to have a strong affinity for NMDARs, even greater than that of (R,S)-ketamine and (S)-ketamine. Its inhibitor constant (Ki) is 1.7 μM compared with 0.53 μM and 0.3 μM, respectively. This metabolite has demonstrated rapid and powerful antidepressant effects in rodent studies. While preclinical research has highlighted the potential for esketamine abuse, (S)-norketamine appears to carry a lower risk of psychotomimetic effects and addiction, making it a safer alternative [55]. The study suggested that activation of AMPARs is not required for the antidepressant effects of (S)-norketamine, as AMPAR antagonists did not block its effects. Instead, the antidepressant’s action appears to involve the BDNF, tropomyosin kinase B (TrkB), and mTORC signaling pathways [41,56]. However, recent clinical evidence found no correlation between norketamine levels—whether (S)-norketamine or (R)-norketamine—and an antidepressant response following the administration of (R,S)-ketamine in patients with treatment-resistant depression [41,57].

(S)-ketamine, recognized for its higher affinity for NMDARs, was investigated as a novel antidepressant by Janssen Research & Development. In an initial trial, intravenous (S)-ketamine at doses of 0.2 mg/kg and 0.4 mg/kg produced rapid and strong antidepressant effects in individuals with TRD. Side effects included headache, nausea, and dissociation. As the antidepressant benefits were similar between both doses, it was suggested that a lower dose could provide better tolerability without sacrificing effectiveness. A fixed-dose (S)-ketamine nasal spray was later developed and tested in TRD patients. Several Phase II and III trials showed that combining intranasal (S)-ketamine with an oral antidepressant was more effective than a placebo combined with oral antidepressants [58,59,60,61], though some studies did not show positive results [62,63]. A large study with 297 TRD patients found that continuing the (S)-ketamine nasal spray treatment delayed the time to relapse compared with a placebo after 16 weeks of treatment. An open-label study explored the long-term safety of (S)-ketamine nasal spray with an oral antidepressant, showing that common side effects such as dizziness, dissociation, nausea, and headache were mild and temporary, and declined with continued use. Cognitive performance either improved or remained stable over time. Such long-term safety data are not yet available for other forms of ketamine. According to the available evidence, the FDA and EMA approved the (S)-ketamine nasal spray Spravato for adults with TRD when combined with an oral antidepressant. However, concerns about its efficacy, safety, and abuse potential, and the need for careful monitoring still limit its broader use [41].

Administration of S-ketamine increases muscle tone and saliva production while preserving the functionality of reflexes such as swallowing, blinking, coughing, and gagging. Cardiovascular effects include a dose-dependent stimulation of the sympathetic nervous system, leading to increased heart rate, blood pressure, and cardiac output, though peripheral vascular resistance remains relatively unchanged. At high doses or with rapid administration, there may be a slight suppression of breathing and increased mucus production. S-ketamine can also cause bronchodilation through its action on L-type calcium channels and has been noted for its anti-inflammatory effects, which may contribute to its ability to reduce pain sensitivity. The impact of these anti-inflammatory effects in clinical practice is still debated, although experimental data support their significance. Research has shown that S-ketamine affects cerebral blood flow and can increase intracranial pressure, especially in patients with severe brain injury, if not carefully managed with normoventilation. Psychotomimetic effects are uncommon at lower doses (0.125–0.25 mg/kg) but can occur in up to 12% of patients at higher doses. At anesthetic doses (0.5–1 mg/kg), S-ketamine induces dissociative anesthesia, characterized by catalepsy and analgesia, with some patients experiencing open eyes and spontaneous movements, yet retaining some reflexes. While patients may have vivid or unpleasant dreams, these effects are less frequent compared with the racemate and can be mitigated with medications such as propofol or midazolam. Common side effects include nausea, vomiting, dizziness, and impaired vision, which can generally be managed with adjunctive medications such as 5-hydroxytryptamine type 3 (5-HT_3_) receptor antagonists or dimenhydrinate. The exact mechanism behind these side effects is not fully understood but may involve interactions with serotonin receptors [33,64].

The risk of dependence and the possibility of misuse associated with ketamine, especially in its esketamine form, pose significant challenges that have restricted its widespread use in clinical settings, despite its promising effectiveness in managing severe and treatment-resistant depression. The dissociative properties of ketamine and its association with recreational “club drugs” have generated concerns about potential abuse and addiction, particularly when not administered in controlled environments. These risks are heightened by the short-lived nature of its antidepressant effects, which may lead patients to pursue more frequent doses for symptom relief, thereby increasing the likelihood of dependence. Moreover, temporary side effects such as neurocognitive disturbances and variations in heart rate and blood pressure might discourage healthcare providers from prescribing it, particularly for populations at a risk of substance use disorders. Regulatory agencies such as the FDA and EMA have implemented strict monitoring guidelines for esketamine to address these concerns, requiring supervised administration and diligent patient oversight, which further limit its accessibility.

## 4. Arketamine

(R)-(-)-ketamine or (R)-ketamine, also known as arketamine, may serve as a fast-acting antidepressant. Although (R)-ketamine is less potent than (R,S)-ketamine in inhibiting NMDARs in laboratory settings, the degree to which (R)-ketamine produces NMDA receptor-related side effects similar to (R,S)-ketamine in living organisms has not been fully studied. Additionally, (R)-ketamine is metabolized into HNK, which may play a role in its antidepressant effects [65].

Despite having a four times lower affinity for NMDARs than (S)-ketamine, (R)-ketamine shows stronger and more prolonged antidepressant effects in rodents, with fewer psychomotor side effects and a lower potential for abuse. It also surpasses (R,S)-ketamine and the NMDAR antagonist lanicemine, also known as AZD6765 or AR-R 15896AR (a low-trapping NMDA channel blocker), in producing long-lasting antidepressant effects without significantly increasing the release of dopamine in the medial prefrontal cortex (mPFC). Research has suggested that the antidepressant effects of (R)-ketamine may not be linked to NMDAR blockade, lateral habenula (LHb) activity, or dopamine receptor activation. Further studies have shown that blocking AMPARs, transforming growth factor-β1 (TGF-β1) signaling, colony-stimulating factor 1 (CSF1R), and GABA receptors (GABARs) inhibits (R)-ketamine’s antidepressant effects. This indicates that the activation of TGF-β1, CSF1R, and AMPAR, and GABAAR inhibition are vital for its rapid and sustained antidepressant actions. In comparison with (S)-ketamine, (R)-ketamine’s prolonged effects may involve the nuclear receptor binding protein 1 (NRBP1) in microglial cells of the mPFC. It enhances NRBP1, BDNF, and phosphorylated cAMP-responsive element binding protein (p-CREB/CREB) levels, contributing to its long-lasting antidepressant outcomes [66].

(R)-ketamine, through the BDNF-TrkB signaling pathway, helps restore reduced BDNF levels in key brain regions such as the prefrontal cortex (PFC), hippocampal region CA3, and dentate gyrus (DG) in rodents. It also increases serotonin (5-HT) release in the mPFC and inhibits the overexpression of the nuclear factor of activated T-cells and the cytoplasmic 4 (NFATc4) gene in the PFC, highlighting the importance of BDNF-TrkB, NFATc4 signaling, and 5-HT receptors in its antidepressant effects. Additionally, the activation of mTOR and extracellular signal-regulated kinase (ERK) has been suggested as a potential mechanism of ketamine’s effects, although studies have shown mixed results, depending on the depression model used. For instance, in a chronic social defeat stress (CSDS) model, (R)-ketamine reversed reductions in ERK signaling but had no effect on mTOR, while mTOR inhibitors did not block its antidepressant effects. Conversely, in a chronic mild stress (CUMS) model, (R)-ketamine increased mTOR signaling without affecting ERK. These differences suggest that various experimental factors influence the outcomes and further investigation is needed. Furthermore, (R)-ketamine has shown benefits in reducing depressive symptoms by targeting miRNAs, particularly miR-132-5p, and related genes such as BDNF and methyl CpG binding protein 2 (MeCP2). It also has a mild impact on endoplasmic reticulum (ER) stress genes, suggesting that the unfolded protein response (UPR) and the basic leucine zipper transmembrane transcription factor localized in the endoplasmic reticulum (ER) that is cleaved in its transmembrane region in response to the ER stress—OASIS family may play a role in its antidepressant effects. More research is needed to clarify the exact mechanisms involved [67]. (R)-ketamine has demonstrated anti-inflammatory properties and can reduce spleen weight in mice susceptible to chronic social defeat stress (CSDS). This improvement is linked to reduced expression of the natural killer cell receptor (NKG2D) in the spleen. Additionally, (R)-ketamine partially restores changes in the gut microbiota, suggesting that its antidepressant effects may involve both the brain–spleen and microbiota–gut–brain axes. While the metabolite (2R, 6R)-HNK has been shown to have rapid and lasting antidepressant effects in some animal studies, not all researchers have observed similar results. Some argued that (R)-ketamine’s antidepressant effects are independent of (2R, 6R)-HNK, which is considered a pharmacologically inert molecule with weak interactions across different biological systems. The precise role of (2R, 6R)-HNK in (R)-ketamine’s antidepressant action remains uncertain and requires further investigation. Although preclinical research has explored various mechanisms behind (R)-ketamine’s antidepressant effects, the exact pathways and target sites are still not fully understood. However, these findings provide valuable insights for future studies and potential clinical applications [67].

Currently, no (R)-ketamine drug formulations have been approved for market use, but clinical research continues to assess its antidepressant efficacy and safety. A study by Leal et al. [68] documented a single intravenous infusion of (R)-ketamine (0.5 mg/kg) in seven patients with TRD. They observed rapid and significant antidepressant effects, with improvements starting 60 min post-infusion, peaking at 240 min, and lasting in 43% of participants for up to 7 days. Mild side effects such as blurred vision and dizziness were reported, but there were no instances of dissociation or hemodynamic issues, suggesting good safety. However, the open-label design of the study limited the strength of the findings. To address this, the researchers conducted a randomized, double-blind crossover pilot trial involving 10 patients. Over two weeks, both (R)-ketamine and saline were tested. While depressive symptoms improved over time, there was no significant difference between the (R)-ketamine and saline groups, raising questions about its antidepressant efficacy. Participants in the second study had longer histories of depression and more psychiatric comorbidities, which might explain the results. Previous studies have suggested that some patients may require multiple doses for a response to its application [67].

A single administration of (R)-ketamine may not be sufficient to achieve the desired antidepressant effects, which may require cumulative dosing. Additionally, the crossover design used in some studies may not be ideal, as the optimal dosage and frequency for (R)-ketamine treatment are still uncertain. Given that efficacy against depression is often measured by Montgomery–Åsberg Depression Rating Scale (MADRS) scores, detecting significant differences in small groups (such as the 10-patient sample in Leal et al.’s [69] study) is challenging. Although this pilot study did not show that a single infusion of (R)-ketamine was more effective than a placebo in treating depression, it did not completely rule out its potential antidepressant effects. Moreover, (R)-ketamine has shown promising safety and minimal side effects. Other recent research has further supported its antidepressant efficacy, particularly in treating bipolar depression. For instance, a study with six bipolar disorder patients (Types I and II) who received intravenous (R)-ketamine at doses of 0.5 mg/kg and 1 mg/kg a week apart showed favorable results. In the study, the participants’ average MADRS scores dropped by over 50% after treatment, and there were minimal dissociative or manic symptoms at both doses. This indicates that (R)-ketamine is both effective and safe for its rapid antidepressant effects in treating bipolar depression [70]. These findings highlight the potential of (R)-ketamine as a promising antidepressant. Future research will need larger sample sizes, flexible dosing schedules, and alternative study designs, such as parallel subgroup approaches, to better understand its true antidepressant effectiveness in clinical practice [63]. In 2018, China registered a large randomized controlled trial to compare the safety and effectiveness of (R)-ketamine with (S)-ketamine and (R,S)-ketamine for treating TRD [71]. On February 19, 2021, the American company Perception Neuroscience released Phase I data showing that higher doses of (R)-ketamine (PCN-101) are required to cause perceptual changes compared with (S)-ketamine, with doses below 150 mg being safe and well-tolerated. The company has also launched a Phase II trial to further evaluate the therapeutic effects and side effects of (R)-ketamine in TRD patients [72]. In preclinical studies, there has been debate about the antidepressant effectiveness of (2R, 6R)-HNK, a metabolite of (R)-ketamine. Grunebaum et al. [73] found that although patients with major depressive disorder (MDD) and suicidal ideation had higher plasma levels of (2R, 6R)-HNK 24 h after receiving intravenous ketamine (0.5 mg/kg), this did not correlate with significant clinical improvements in depression. This finding suggests caution when interpreting the antidepressant effects of (2R, 6R)-HNK. A Phase I clinical trial is currently in progress to better assess the antidepressant potential of the drug [74].

While (R)-ketamine has demonstrated notable benefits in animal models of depression, its antidepressant effects in clinical settings remain uncertain. To better understand its true efficacy, along with the safety, potential for drug resistance, side effects, and abuse risks associated with medium- to long-term high-dose use, further research is needed. This will involve large-scale, multi-center, double-blind randomized controlled trials that examine various dosing regimens, frequencies, and treatment schedules [67].

**Figure 3:**
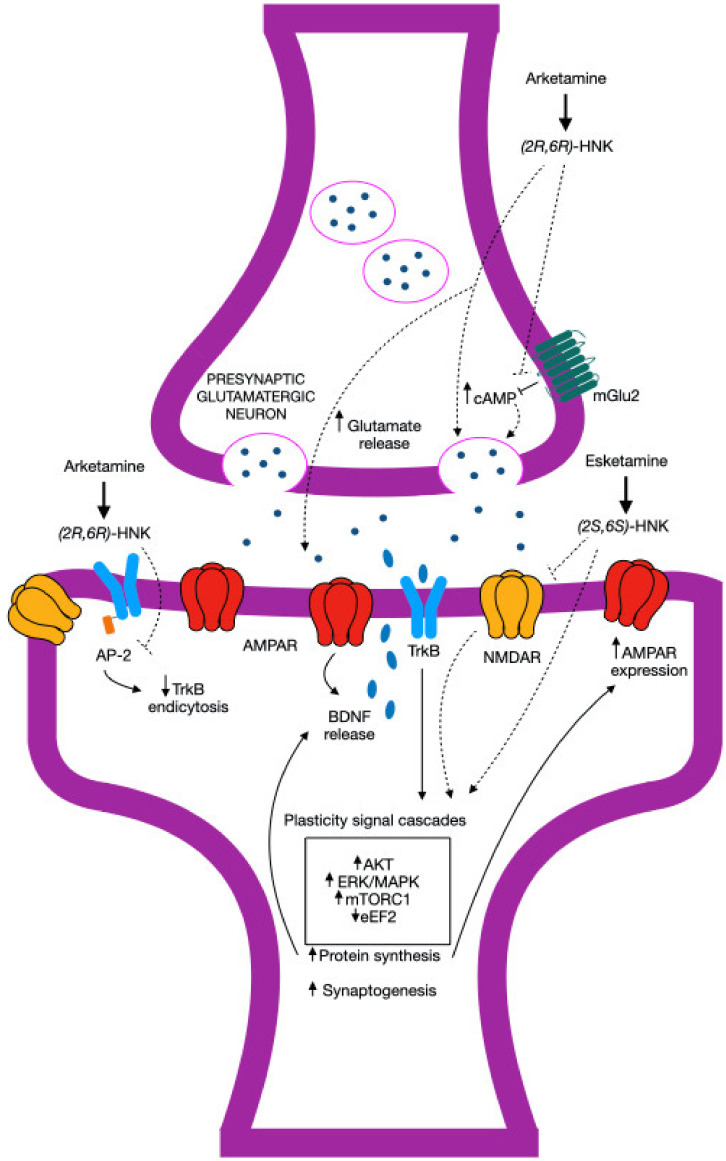
Possible synaptic mechanisms of (2R,6R)- and (2S,6S)-hydroxynorketamine, according to [130]: (2R,6R)-HNK is believed to act on presynaptic terminals by increasing glutamate release, potentially through pathways that overlap with mGlu_2_ signaling. This may occur as (2R,6R)-HNK reduces the inhibition of cAMP release induced by mGlu_2_, or it may involve another mechanism driving glutamate release. The increased glutamate subsequently activates AMPA receptors (AMPAR), leading to the enhanced release of brain-derived neurotrophic factor (BDNF), activation of tropomyosin kinase B (TrkB) receptors, and the triggering of plasticity-related signaling pathways. These pathways include the upregulation of protein kinase B (AKT), extracellular signal-regulated kinases (ERK)/mitogen-activated protein kinases (MAPK), and mammalian/mechanistic target of rapamycin complex 1 (mTORC1), all of which promote protein synthesis, increase AMPAR expression, and support synapse formation, ultimately strengthening synaptic connections. Additionally, (2R,6R)-HNK may interfere with TrkB/AP-2 (Activator Protein-2) interactions, preventing TrkB endocytosis and stabilizing TrkB at the synapse. On the other hand, (2S,6S)-HNK moderately inhibits NMDA receptors (NMDARs) and might enhance intracellular signaling through an NMDAR inhibition-dependent mechanism, which includes the inhibition of eEF2 signaling, alongside increased AKT, ERK/MAPK, and mTORC1 activity.

Figure 3 presents the proposed synaptic mechanisms of (2R,6R)- and (2S,6S)-hydroxynorketamine, while Table 2 provides Supplementary Information on studies involving (R)-ketamine in nondepressive conditions.

**Table 2:** Research into the use of (R)-ketamine beyond depression, according to [67].

Condition	References
Cognitive impairments	[16,34,75,76,77,78,79,80,81,82,83,84,85,86]
COVID-19	[87,88,89,90,91,92,93]
Inflammatory disease	[36,37,78,91,94,95,96,97,98]
Ischemic stroke	[99,100,101,102,103]
Multiple sclerosis	[48,92]
Organophosphate poisoning	[104,105,106,107,108]
Osteoporosis	[109,110,111,112,113]
Parkinson’s disease	[114,115,116,117,118]
Perioperative anesthesia	[13,96,119,120,121,122,123,124,125,126]
Substance use disorder	[127,128,129]

## 5. Conclusions

Ketamine’s discovery has shifted the focus from slow-acting monoaminergic systems to fast glutamatergic neurotransmission in mood regulation, opening new research avenues in the treatment of depression. The drug’s ability to induce homeostatic plasticity suggests it provides temporary symptom relief rather than addressing depression’s underlying causes. Future research aims to extend the therapeutic benefits of ketamine by targeting the downstream pathways, potentially reducing the need for repeated doses and mitigating the side effects. The potential for new rapid-acting antidepressants with fewer risks and broader applications is significant, but ongoing research is required to validate these strategies and better understand ketamine’s mechanisms of action. Esketamine’s ability to rapidly relieve depressive symptoms and lessen suicidal thoughts has made it a crucial option for treating TRD. However, it requires strict monitoring due to potential side effects and the risk of misuse. Its approval comes with stringent guidelines for administration and supervision. Arketamine, with fewer side effects and possibly more potent antidepressant effects than esketamine, shows promise but requires further research to verify its long-term safety and effectiveness. While esketamine is already in clinical use, arketamine’s future depends on additional trials to resolve outstanding concerns about its safety, efficacy, and appropriate dosing.

## Figures and Tables

**Table 1:** Comparison of antidepressant and side effects of racemic ketamine, esketamine, and arketamine in humans and animals, according to [43].

**Antidepressant effects—animal studies**
Antidepressant effects: arketamine > racemic ketamine and esketamine
Racemic ketamine, esketamine, and arketamine
Decrease in immobility time in the forced swim test (FST)/or tail suspension test TST
Increase in sucrose preference in the sucrose preference test (SPT)
**Side effects—animal studies**
Side effects: arketamine < racemic ketamine and esketamine
Racemic ketamine and esketamine
Hyperlocomotion
Psychomimetic effects
Rewarding effects
Abuse liability
Arketamine
Mild effects on locomotion
Cognitive process profile (CPP) scores, motor coordinator deficits, and prepulse inhibition (PPI)
No serious adverse events were reported
**Antidepressant effects—humans**
Racemic ketamine, esketamine, and arketamine
Reduced score on the Montgomery–Åsberg Depression Rating Scale (MADRS)/Hamilton Depression Rating Scale (HDRS)
Ketamine therapy includes, among others, depression (even treatment-resistant), anxiety, suicidal ideation, post-traumatic stress disorder (PTSD), obsessive–compulsive disorder (OCD), neuropathic pain, chronic pain, substance abuse and eating disorders; esketamine: treatment-resistant depression and major depressive disorder with acute suicidal ideation or behavior
**Side effects—humans**
Racemic ketamine and esketamine
Headache
Dizziness
Dissociation
Rewarding effects
Abuse liability
Cognitive dysfunction
Arketamine
No serious adverse events were reported

## Data Availability

Data sharing is not applicable to this article.

## Supplementary Materials

The following supporting information can be downloaded at: https://www.mdpi.com/article/10.3390/biomedicines12102283/s1.

## References

[1] Ball C., Westhorpe R. (2002). Intravenous Induction Agents: Ketamine. Anaesth. Intensive Care.

[2] Mion G. (2017). History of anaesthesia The ketamine story—Past, present and future. Eur. J. Anaesthesiol..

[3] Denomme N.B.S. (2018). The Domino Effect: Ed Domino’s early studies of Psychoactive Drugs. J. Psychoact. Drugs.

[4] Das J. (2020). Repurposing of Drugs-The Ketamine Story. J. Med. Chem..

[5] Bloomfield A., Chan N., Fryml L., Horace R., Pyati S. (2023). Ketamine for Chronic Pain and Mental Health: Regulations, Legalities, and the Growth of Infusion Clinics. Curr. Pain Headache Rep..

[6] Shi X., Zhou J., Jiang H., Xie K. (2024). Ketamine in the Management of Acute Pain: A Comprehensive Meta-Analysis. J. Coll. Physicians Surg. Pak..

[7] Merelman A.H., Perlmutter M.C., Strayer R.J. (2019). Alternatives to Rapid Sequence Intubation: Contemporary Airway Management with Ketamine. W. J. Emerg. Med..

[8] Nichols K.A., Paciullo C.A. (2019). Subdissociative Ketamine Use in the Emergency Department. Adv. Emerg. Nurs. J..

[9] Mashour G.A. (2024). Ketamine and the paradox of anaesthetic state transitions. Br. J. Anaesth..

[10] Tian F., Lewis L.D., Zhou D.W., Balanza G.A., Paulk A.C., Zelmann R., Peled N., Soper D., Santa Cruz Mercado L.A., Peterfreund R.A. (2023). Characterizing brain dynamics during ketamine-induced dissociation and subsequent interactions with propofol using human intracranial neurophysiology. Nat. Commun..

[11] McMurray M., Orthober R., Huecker M. (2024). Ketamine’s love story with the heart: A Takotsubo twist. Am. J. Emerg. Med..

[12] Reich D.L., Silvay G. (1989). Ketamine: An update on the first twenty-five years of clinical experience. Can. J. Anaesth..

[13] Barrett W., Buxhoeveden M., Dhillon S. (2020). Ketamine: A versatile tool for anesthesia and analgesia. Curr. Opin. Anaesthesiol..

[14] Nowacka A., Borczyk M. (2019). Ketamine applications beyond anesthesia—A literature review. Eur. J. Pharmacol..

[15] Wilkowska A., Wiglusz M.S., Jakuszkowiak-Wojten K., Cubała W.J. (2022). Ketamine and Lamotrigine Combination in Psychopharmacology: Systematic Review. Cells.

[16] Passie T., Adams H.A., Logemann F., Brandt S.D., Wiese B., Karst M. (2021). Comparative effects of (S)-ketamine and racemic (R/S)-ketamine on psychopathology, state of consciousness and neurocognitive performance in healthy volunteers. Eur. Neuropsychopharmacol..

[17] Feder A., Rutter S.B., Schiller D., Charney D.S. (2020). The emergence of ketamine as a novel treatment for posttraumatic stress disorder. Adv. Pharmacol..

[18] Zanos P., Moaddel R., Morris P.J., Riggs L.M., Highland J.N., Georgiou P., Pereira E.F.R., Albuquerque E.X., Thomas C.J., Zarate C.A. (2018). Ketamine and Ketamine Metabolite Pharmacology: Insights into Therapeutic Mechanisms. Pharmacol. Rev..

[19] Fukumoto K., Duman R.S., Hashimoto K., Manto M. (2021). (2R,6R)-Hydroxynorketamine, a Metabolite of Ketamine: The Antidepressant Actions and the Mechanisms. New Rapid-Acting Antidepressants.

[20] Schwenk E.S., Pradhan B., Nalamasu R., Stolle L., Wainer I.W., Cirullo M., Olson A., Pergolizzi J.V., Torjman M.C., Viscusi E.R. (2021). Ketamine in the Past, Present, and Future: Mechanisms, Metabolites, and Toxicity. Curr. Pain Headache Rep..

[21] Kadriu B., Ballard E.D., Henter I.D., Murata S., Gerlus N., Zarate C.A. (2020). Neurobiological biomarkers of response to ketamine. Adv. Pharmacol..

[22] Marguilho M., Figueiredo I., Castro-Rodrigues P. (2023). A unified model of ketamine’s dissociative and psychedelic properties. J. Psychopharmacol..

[23] Adams H.A., Werner C. (1997). From the racemate to the eutomer: (S)-ketamine. Renaissance of a substance?. Anaesthesist.

[24] Lii T.R., Singh V. (2023). Ketamine for Complex Regional Pain Syndrome: A Narrative Review Highlighting Dosing Practices and Treatment Response. Anesthesiol. Clin..

[25] Kohtala S. (2021). Ketamine-50 years in use: From anesthesia to rapid antidepressant effects and neurobiological mechanisms. Pharmacol. Rep..

[26] Wei Y., Chang L., Hashimoto K. (2020). A historical review of antidepressant effects of ketamine and its enantiomers. Pharmacol. Biochem. Behav..

[27] Himmelseher S., Kochs E.F. (2021). Ready for a “breakthrough” with ketamine? A look at recent pharmacological insights!. Curr. Opin. Anaesthesiol..

[28] Schatzberg A.F. (2021). Mechanisms of Action of Ketamine and Esketamine. Am. J. Psychiatry.

[29] Kheirkhah M., Nugent A.C., Livinski A.A., Neely L., Johnson S.C., Henter I.D., Varnosfaderani S.D., Price R.B., Hejazi N., Yavi M. (2024). Exploring the impact of music on response to ketamine/esketamine: A scoping review. Neurosci. Biobehav. Rev..

[30] Kalkman H.O. (2023). Activation of σ1-Receptors by R-Ketamine May Enhance the Antidepressant Effect of S-Ketamine. Biomedicines.

[31] Feeney A., Papakostas G.I. (2023). Pharmacotherapy: Ketamine and Esketamine. Psychiatr. Clin. N. Am..

[32] Zhang K., Hashimoto K. (2019). An update on ketamine and its two enantiomers as rapid-acting antidepressant. Expert Rev. Neurother..

[33] Trimmel H., Helbok R., Staudinger T., Jaksch W., Messerer B., Schöchl H., Likar R. (2018). S(+)-ketamine: Current trends in emergency and intensive care medicine. Wien. Klin. Wochenschr..

[34] Yokoyama R., Ago Y., Igarashi H., Higuchi M., Tanuma M., Shimazaki Y., Kawai T., Seiriki K., Hayashida M., Yamaguchi S. (2024). (R)-ketamine restores anterior insular cortex activity and cognitive deficits in social isolation-reared mice. Mol. Psychiatry.

[35] Brown G.C. (2019). The endotoxin hypothesis of neurodegeneration. J. Neuroinflamm..

[36] Ma L., Zhang J., Fujita Y., Qu Y., Shan J., Wan X., Wang X., Ishima T., Kobayashi K., Wang L. (2022). Nuclear factor of activated T cells 4 in the prefrontal cortex is required for prophylactic actions of (R)-ketamine. Transl. Psychiatry.

[37] Fujita Y., Hashimoto Y., Hashimoto H., Chang L., Hashimoto K. (2021). Dextran sulfate sodium-induced inflammation and colitis in mice are ameliorated by (R)-ketamine but not (S)-ketamine: A role of TrkB signaling. Eur. J. Pharmacol..

[38] Zhang K., Sakamoto A., Chang L., Qu Y., Wang S., Pu Y., Tan Y., Wang X., Fujita Y., Ishima T. (2021). Splenic NKG2D confers resilience versus susceptibility in mice after chronic social defeat stress: Beneficial effects of (R)-ketamine. Eur. Arch. Psychiatry Clin. Neurosci..

[39] Hashimoto K. (2020). Molecular mechanisms of the rapid-acting and long-lasting antidepressant actions of (R)-ketamine. Biochem. Pharmacol..

[40] Hashimoto K. (2020). Are NMDA and opioid receptors involved in the antidepressant actions of ketamine?. Proc. Natl. Acad. Sci. USA.

[41] Jelen L.A., Young A.H., Stone J.M. (2021). Ketamine: A tale of two enantiomers. J. Psychopharmacol..

[42] Ma L., Hashimoto K. (2022). The role of hippocampal KCNQ2 channel in antidepressant actions of ketamine. Neuron.

[43] Scotton E., Antqueviezc B., Vasconcelos M.F., Dalpiaz G., Géa L.P., Ferraz Goularte J., Colombo R., Ribeiro Rosa A. (2022). Is (R)-ketamine a potential therapeutic agent for treatment-resistant depression with less detrimental side effects? A review of molecular mechanisms underlying ketamine and its enantiomers. Biochem. Pharmacol..

[44] Wei Y., Chang L., Hashimoto K. (2022). Molecular mechanisms underlying the antidepressant actions of arketamine: Beyond the NMDA receptor. Mol. Psychiatry.

[45] Rickards H. (2005). Depression in neurological disorders: Parkinson’s disease, multiple sclerosis, and stroke. J. Neurol. Neurosurg. Psychiatry.

[46] Hesdorffer D.C. (2016). Comorbidity between neurological illness and psychiatric disorders. CNS Spectr..

[47] Wang X., Yang J., Hashimoto K. (2022). (R)-ketamine as prophylactic and therapeutic drug for neurological disorders: Beyond depression. Neurosci. Biobehav. Rev..

[48] Wang X., Chang L., Wan X., Tan Y., Qu Y., Shan J., Yang Y., Ma L., Hashimoto K. (2022). (R)-ketamine ameliorates demyelination and facilitates remyelination in cuprizone-treated mice: A role of gut-microbiota-brain axis. Neurobiol. Dis..

[49] Nikolin S., Rodgers A., Schwaab A., Bahji A., Zarate C.A., Vazquez G., Loo C. (2023). Ketamine for the treatment of major depression: A systematic review and meta-analysis. eClinicalMedicine.

[50] Meshkat S., Haikazian S., Di Vincenzo J.D., Fancy F., Johnson D., Chen-Li D., McIntyre R.S., Mansur R., Rosenblat J.D. (2023). Oral ketamine for depression: An updated systematic review. World J. Biol. Psychiatry.

[51] Mandal S., Sinha V.K., Goyal N. (2019). Efficacy of ketamine therapy in the treatment of depression. Indian J. Psychiatry.

[52] Aleksandrova L.R., Phillips A.G., Yu Wang Y.T. (2017). Antidepressant effects of ketamine and the roles of AMPA glutamate receptors and other mechanisms beyond NMDA receptor antagonism. J. Psychiatry Neurosci..

[53] Krystal J.K., Kavalali E.T., Monteggia L.M. (2024). Ketamine and rapid antidepressant action: New treatments and novel synaptic signaling mechanisms. Neuropsychopharmacology.

[54] Sanacora G., Schatzberg A.F. (2015). Ketamine: Promising path or false prophecy in the development of novel therapeutics for mood disorders?. Neuropsychopharmacology.

[55] Vasiliu O. (2023). Esketamine for treatment-resistant depression: A review of clinical evidence (Review). Exp. Ther. Med..

[56] Yang C., Kobayashi S., Nakao K., Dong C., Han M., Qu Y., Ren Q., Zhang J.-C., Ma M., Toki H. (2018). AMPA receptor activation-independent antidepressant actions of ketamine metabolite (S)-norketamine. Biol. Psychiatry.

[57] Farmer C.A., Gilbert J.R., Moaddel R., George J., Adeojo L., Lovett J., Nugent A.C., Kadriu B., Yuan P., Gould T.D. (2020). Ketamine metabolites, clinical response, and gamma power in a randomized, placebo-controlled, crossover trial for treatment-resistant major depression. Neuropsychopharmacology.

[58] Canuso C.M., Singh J.B., Fedgchin M., Alphs L., Lane R., Lim P., Pinter C., Hough D., Sanacora G., Manji H. (2018). Efficacy and safety of intranasal esketamine for the rapid reduction of symptoms of depression and suicidality in patients at imminent risk for suicide: Results of a double-blind, randomized, placebo-controlled study. Am. J. Psychiatry.

[59] Daly E.J., Singh J.B., Fedgchin M., Cooper K., Lim P., Shelton R.C., Thase M.E., Winokur A., Van Nueten L., Manji H. (2018). Efficacy and safety of intranasal esketamine adjunctive to oral antidepressant therapy in treatment-resistant depression: A randomized clinical trial. JAMA Psychiatry.

[60] Daly E.J., Trivedi M.H., Janik A., Li H., Zhang Y., Li X., Lane R., Lim P., Duca A.R., Hough D. (2019). Efficacy of esketamine nasal spray plus oral antidepressant treatment for relapse prevention in patients with treatment-resistant depression: A randomized clinical trial. JAMA Psychiatry.

[61] Popova V., Daly E.J., Trivedi M., Cooper K., Lane R., Lim P., Mazzucco C., Hough D., Thase M.E., Shelton R.C. (2019). Efficacy and safety of flexibly dosed esketamine nasal spray combined with a newly initiated oral antidepressant in treatment-resistant depression: A randomizeddouble-blind active-controlled study. Am. J. Psychiatry.

[62] Fedgchin M., Trivedi M., Daly E.J., Melkote R., Lane R., Lim P., Vitagliano D., Blier P., Fava M., Liebowitz M. (2019). Efficacy and safety of fixed-dose esketamine nasal spray combined with a new oral antidepressant in treatment-resistant depression: Results of a randomized, double-blind, active-controlled study (TRANSFORM-1). Int. J. Neuropsychopharmacol..

[63] Ochs-Ross R., Daly E.J., Zhang Y., Lane R., Lim P., Morrison R.L., Hough D., Manji H., Drevets W.C., Sanacora G. (2020). Efficacy and safety of esketamine nasal spray plus an oral antidepressant in elderly patients with treatment-resistant depression—TRANSFORM-3. Am. J. Geriatr. Psychiatry.

[64] Annetta M.G., Iemma D., Garisto C., Tafani C., Proietti R. (2005). Ketamine: New indications for an old drug. Curr. Drug Targets.

[65] He T., Wang D., Wu Z., Huang C., Xu X., Xu X., Liu C., Hashimoto K., Yang C. (2022). A bibliometric analysis of research on (R)-ketamine from 2002 to 2021. Neuropharmacology.

[66] Zanos P., Highland J.N., Liu X., Troppoli T.A., Georgiou P., Lovett J., Morris P.J., Stewart B.W., Thomas C.J., Thompson S.M. (2019). (R)-Ketamine exerts antidepressant actions partly via conversion to (2R,6R)-hydroxynorketamine, while causing adverse effects at sub-anaesthetic doses. Br. J. Pharmacol..

[67] Zhang S., Pu Y., Liu J., Li L., An C., Wu Y., Zhang W., Zhang W., Qu S., Yan W. (2024). Exploring the multifaceted potential of (R)-ketamine beyond antidepressant applications. Front. Pharmacol..

[68] Leal G., Bandeira I., Correia-Melo F., Telles M., Mello R., Vieira F., Lima C.S., Jesus-Nunes A.P., Guerreiro-Costa L.N.F., Marback R.F. (2021). Intravenous arketamine for treatment-resistant depression: Open-label pilot study. Eur. Arch. Psychiatry Clin. Neurosci..

[69] Leal G.C., Souza-Marques B., Mello R.P., Bandeira I.D., Caliman-Fontes A.T., Carneiro B.A., Faria-Guimarães D., Guerreiro-Costa L.N.F., Jesus-Nunes A.P., Silva S.S. (2023). Arketamine as adjunctive therapy for treatment-resistant depression: A placebo-controlled pilot study. J. Affect. Disord..

[70] Bandeira I.D., Leal G.C., Correia-Melo F.S., Souza-Marques B., Silva S.S., Lins-Silva D.H., Mello R.P., Vieira F., Dorea-Bandeira I., Faria-Guimarães D. (2023). Arketamine for bipolar depression: Open-label, dose-escalation, pilot study. J. Psychiatr. Res..

[71] Chinese Clinical Trial Registry Efficacy and Safety of Ketamine, S-Ketamine and R-Ketamine in Treatment Resistant Depression: A Randomized Controlled Trial. https://www.chictr.org.cn/showproj.html?proj=26844.

[72] PR Newswire Perception Neuroscience’s PCN-101 (R-Ketamine) Demonstrates Tolerability in Phase 1 Single Ascending Dose Study. https://www.prnewswire.com/news-releases/perception-neurosciences-pcn-101-r-ketamine-demonstrates-tolerability-in-phase-1-single-ascending-dose-study-301231491.html.

[73] Grunebaum M.F., Galfalvy H.C., Choo T.H., Parris M.S., Burke A.K., Suckow R.F., Cooper T.B., Mann J.J. (2019). Ketamine metabolite pilot study in a suicidal depression trial. J. Psychiatr. Res..

[74] ClinicalTrials Phase 1 Evaluation of (2R,6R)-Hydroxynorketamine. https://clinicaltrials.gov/study/NCT04711005.

[75] Tan Y., Fujita Y., Pu Y., Chang L., Qu Y., Wang X., Hashimoto K. (2022). Repeated intermittent administration of (R)-ketamine during juvenile and adolescent stages prevents schizophrenia-relevant phenotypes in adult offspring after maternal immune activation: A role of TrkB signaling. Eur. Arch. Psychiatry Clin. Neurosci..

[76] Ide S., Ikekubo Y., Mishina M., Hashimoto K., Ikeda K. (2019). Cognitive impairment that is induced by (R)-ketamine is abolished in NMDA GluN2D receptor subunit knockout mice. Int. J. Neuropsychopharmacol..

[77] Tan Y., Fujita Y., Qu Y., Chang L., Pu Y., Wang S., Wang X., Hashimoto K. (2020). Phencyclidine-induced cognitive deficits in mice are ameliorated by subsequent repeated intermittent administration of (R)-ketamine, but not (S)-ketamine: Role of BDNF-TrkB signaling. Pharmacol. Biochem. Behav..

[78] Zhang J., Ma L., Wan X., Shan J., Qu Y., Hashimoto K. (2021). (R)-ketamine attenuates LPS-induced endotoxin-derived deliriumthrough inhibition of neuroinflammation. Psychopharmacology.

[79] Li S.H., Abd-Elrahman K.S., Ferguson S.S.G. (2022). Targeting mGluR2/3 for treatment of neurodegenerative and neuropsychiatric diseases. Pharmacol. Ther..

[80] Pałucha-Poniewiera A., Bobula B., Rafało-Ulińska A. (2023). The antidepressant-like activity and cognitive enhancing effects of the combined administration of (R)-Ketamine and LY341495 in the CUMS model of depression in mice are related to themodulation of excitatory synaptic transmission and LTP in the PFC. Pharmaceuticals.

[81] Pothorszki D., Koncz S., Török D., Papp N., Bagdy G. (2024). Unique effects of (R)-ketamine compared to (S)-ketamine on EEG theta power in rats. Pharmaceuticals.

[82] Popik P., Hogendorf A., Bugno R., Khoo S., Zajdel P., Malikowska-Racia N., Nikiforuk A., Golebiowska J. (2022). Effects of ketamine optical isomers, psilocybin, psilocin and norpsilocin on time estimation and cognition in rats. Psychopharmacology.

[83] Zhao Q., Xiang H., Cai Y., Meng S.S., Zhang Y., aQiu P. (2022). Systematic evaluation of the associations between mental disorders and dementia: An umbrella review of systematic reviews and meta-analyses. J. Affect. Disord..

[84] Sabates J., Chiu W.H., Loi S., Lampit A., Gavelin H.M., Chong T., Launder N., Goh A.M.Y., Brodtmann A., Lautenschlager N. (2024). The associations between neuropsychiatric symptoms and cognition in people with dementia: A systematic review and meta-analysis. Neuropsychol. Rev..

[85] Hashimoto K. (2023). Arketamine for cognitive impairment in psychiatric disorders. Eur. Arch. Psychiatry Clin. Neurosci..

[86] Shafique H., Demers J.C., Biesiada J., Golani L.K., Cerne R., Smith J.L., Szostak M., Witkin J.M. (2024). (R)-(-)-Ketamine: The Promise of a Novel Treatment for Psychiatric and Neurological Disorders. Int. J. Mol. Sci..

[87] Robson M.J., Elliott M., Seminerio M.J., Matsumoto R.R. (2012). Evaluation of sigma (σ) receptors in the antidepressant-like effects of ketamine in vitro and in vivo. Eur. Neuropsychopharmacol..

[88] Ortoleva J. (2020). Consider Adjunctive Ketamine in mechanically ventilated coronavirus disease-2019 Patients. J. Cardiothorac. Vasc. Anesth..

[89] Akinosoglou K., Gogos A., Papageorgiou C., Angelopoulos E., Gogos C. (2021). Ketamine in COVID-19 patients: Thinking out of the box. J. Med. Virol..

[90] Hashimoto K. (2021). Repurposing of CNS drugs to treat COVID-19 infection: Targeting the sigma-1 receptor. Eur. Arch. Psychiatry Clin. Neurosci..

[91] Zhang J., Ma L., Hashimoto Y., Wan X., Shan J., Qu Y., Hashimoto K. (2021). (R)-ketamine ameliorates lethal inflammatory responses and multi-organ injury in mice induced by cecum ligation and puncture. Life Sci..

[92] Wang X., Chang L., Tan Y., Qu Y., Shan J., Hashimoto K. (2021). (R)-ketamine ameliorates the progression of experimental autoimmune encephalomyelitis in mice. Brain Res. Bull..

[93] Vollenweider F., Leenders K., Oye I., Hell D., Angst J. (1997). Differential psychopathology and patterns of cerebral glucose utilization produced by (S)- and (R)-ketamine in healthy volunteers using positron emission tomography (PET). Eur. Neuropsychopharmacol..

[94] Lu Y., Ding X., Wu X., Huang S. (2020). Ketamine inhibits LPS-mediated BV2 microglial inflammation via NMDA receptor blockage. Fundam. Clin. Pharmacol..

[95] Zhang Z., Zhang L., Zhou C., Wu H. (2014). Ketamine inhibits LPS-induced HGMB1 release in vitro and in vivo. Int. Immunopharmacol..

[96] Yao W., Cao Q., Luo S., He L., Yang C., Chen J., Qi Q., Hashimoto K., Zhang J.-C. (2022). Microglial ERKNRBP1-CREB-BDNF signaling in sustained antidepressant actions of (R)-ketamine. Mol. Psychiatry.

[97] Frolkis A.D., Vallerand I.A., Shaheen A.A., Lowerison M.W., Swain M.G., Barnabe C., Patten S.B., Kaplan G.G. (2019). Depression increases the risk of inflammatory bowel disease, which may be mitigated by the use of antidepressants in the treatment of depression. Gut.

[98] Zhang X., He T., Wu Z., Wang Y., Liu H., Zhang B., Yang S., Wang D., Huang C., Duan J. (2024). The role of CD38 in inflammation-induced depression-like behavior and the antidepressant effect of (R)-ketamine. Brain Behav. Immun..

[99] Abdoulaye I.A., Wu S.S., Chibaatar E., Yu D.F., Le K., Cao X.J., Guo Y.-J. (2021). Ketamine induces lasting antidepressant effects by modulating the NMDAR/CaMKIImediated synaptic plasticity of the hippocampal dentate gyrus in depressive stroke model. Neural Plast..

[100] Shu L., Li T., Han S., Ji F., Pan C., Zhang B., Li J. (2012). Inhibition of neuronspecific CREB dephosphorylation is involved in propofol and ketamine-induced neuroprotection against cerebral ischemic injuries of mice. Neurochem. Res..

[101] Zhang L.M., Wu Z.Y., Liu J.Z., Li Y., Lv J.M., Wang L.Y., Shan Y.D., Song R.-X., Miao H.-T., Zhang W. (2023). Subanesthetic dose of S-ketamine improved cognitive dysfunction via the inhibition of hippocampal astrocytosis in a mouse model of post-stroke chronic stress. J. Psychiatr. Res..

[102] Johnston J.N., Henter I.D., Zarate C.A. (2023). The antidepressant actions of ketamine and its enantiomers. Pharmacol. Ther..

[103] Xiong Z., Chang L., Qu Y., Pu Y., Wang S., Fujita Y., Ishima T., Chen J., Hashimoto K. (2020). Neuronal brain injury after cerebral ischemic stroke is ameliorated after subsequent administration of (R)-ketamine, but not (S)-ketamine. Pharmacol. Biochem. Behav..

[104] Ribeiro A., Zhu J., Kronfol M., Jahr F., Younis R., Hawkins E., McClay J.L., Deshpande L.S. (2020). Molecular mechanisms for the antidepressant-like effects of a low-dose ketamine treatment in a DFP-based rat model for Gulf War Illness. Neurotoxicology.

[105] Zhu J., Hawkins E., Phillips K., Deshpande L.S. (2020). Assessment of ketamine and its enantiomers in an organophosphate-based rat model for features of Gulf War Illness. Int. J. Environ. Res. Public Health.

[106] Zanos P., Gould T. (2018). Mechanisms of ketamine action as an antidepressant. Mol. Psychiatry.

[107] Ide S., Ikeda K. (2018). Mechanisms of the antidepressant effects of ketamine enantiomers and their metabolites. Biol. Psychiatry.

[108] Ide S., Ikekubo Y., Mishina M., Hashimoto K., Ikeda K. (2017). Role of NMDA receptor GluN2D subunit in the antidepressant effects of enantiomers of ketamine. J. Pharmacol. Sci..

[109] Kadriu B., Gold P., Luckenbaugh D., Lener M., Ballard E., Niciu M.J., Henter I.D., Park L.T., De Sousa R.T., Yuan P. (2018). Acute ketamine administration corrects abnormal inflammatory bone markers in major depressive disorder. Mol. Psychiatry.

[110] Zhang K., Ma M., Dong C., Hashimoto K. (2018). Role of inflammatory bone markers in the antidepressant actions of (R)-ketamine in a chronic social defeat stress model. Int. J. Neuropsychopharmacol..

[111] Xiong Z., Fujita Y., Zhang K., Pu Y., Chang L., Ma M., Chen J., Hashimoto K. (2019). Beneficial effects of (R)-ketamine, but not its metabolite (2R,6R)-hydroxynorketamine, in the depression-like phenotype, inflammatory bone markers, and bone mineral density in a chronic social defeat stress model. Behav. Brain Res..

[112] Wan X., Eguchi A., Fujita Y., Ma L., Wang X., Yang Y., Qu Y., Chang L., Zhang J., Mori C. (2022). Effects of (R)-ketamine on reduced bone mineral density in ovariectomized mice: A role of gut microbiota. Neuropharmacology.

[113] Wan X., Eguchi A., Chang L., Mori C., Hashimoto K. (2023). Beneficial effects of arketamine on the reduced bone mineral density in susceptible mice after chronic social defeat stress: Role of the gut-microbiota-bone-brain axis. Neuropharmacology.

[114] Vecchia D.D., Kanazawa L.K.S., Wendler E., de Almeida Soares Hocayen P., Bruginski E., Campos F.R., Stern C.A.J., Vital M.A.B.F., Miyoshi E., Wöhr M. (2018). Effects of ketamine on vocal impairment, gait changes, and anhedonia induced by bilateral 6-OHDA infusion into the substantia nigra pars compacta in rats: Therapeutic implications for Parkinson’s disease. Behav. Brain Res..

[115] Vecchia D.D., Kanazawa L.K.S., Wendler E., Hocayen P.A.S., Vital M., Takahashi R.N., Da Cunha C., Miyoshi E., Andreatini R. (2021). Ketamine reversed short-term memory impairment and depressive-like behavior in animal model of Parkinson’s disease. Brain Res. Bull..

[116] Fan J.C., Song J.J., Wang Y., Chen Y., Hong D.X. (2017). Neuron-protective effect of subanesthestic-dosage ketamine on mice of Parkinson’s disease. Asian Pac. J. Trop. Med..

[117] Fujita A., Fujita Y., Pu Y., Chang L., Hashimoto K. (2020). MPTP-induced dopaminergic neurotoxicity in mouse brain is attenuated after subsequent intranasal administration of (R)-ketamine: A role of TrkB signaling. Psychopharmacology.

[118] Yang C., Shirayama Y., Zhang J., Ren Q., Yao W., Ma M., Dong C., Hashimoto K. (2015). R-ketamine: A rapid-onset and sustained antidepressant without psychotomimetic side effects. Transl. Psychiatry.

[119] White P., Ham J., Way W., Trevor A. (1980). Pharmacology of ketamine isomers in surgical patients. Anesthesiology.

[120] White P., Schüttler J., Shafer A., Stanski D., Horai Y., Trevor A. (1985). Comparative pharmacology of the ketamine isomers. Studies in volunteers. Br. J. Anaesth..

[121] Olofsen E., Kamp J., Henthorn T., van Velzen M., Niesters M., Sarton E., Dahan A. (2022). Ketamine psychedelic and antinociceptive effects are connected. Anesthesiology.

[122] Geisslinger G., Hering W., Thomann P., Knoll R., Kamp H., Brune K. (1993). Pharmacokinetics and pharmacodynamics of ketamine enantiomers in surgical patients using a stereoselective analytical method. Br. J. Anaesth..

[123] Kamp J., van Velzen M., Aarts L., Niesters M., Dahan A., Olofsen E. (2021). Stereoselective ketamine effect on cardiac output: A population pharmacokinetic/pharmacodynamic modelling study in healthy volunteers. Br. J. Anaesth..

[124] Jonkman K., van der Schrier R., van Velzen M., Aarts L., Olofsen E., Sarton E., Niesters M., Dahan A. (2018). Differential role of nitric oxide in the psychedelic symptoms induced by racemic ketamine and esketamine in human volunteers. Br. J. Anaesth..

[125] de Carvalho C., Lopes M., Constantino L., Hoeller A., de Melo H., Guarnieri R., Linhares M.N., Bortolotto Z.A., Prediger R.D., Latini A. (2021). The ERK phosphorylation levels in the amygdala predict anxiety symptoms in humans and MEK/ERK inhibition dissociates innate and learned defensive behaviors in rats. Mol. Psychiatry.

[126] Yang C., Ren Q., Qu Y., Zhang J., Ma M., Dong C., Hashimoto K. (2018). Mechanistic target of rapamycin-independent antidepressant effects of (R)-ketamine in a social defeat stress model. Biol. Psychiatry.

[127] Jones J.L., Mateus C.F., Malcolm R.J., Brady K.T., Back S.E. (2018). Efficacy of ketamine in the treatment of substance use disorders: A systematic review. Front. Psychiatry.

[128] Witkin J., Kranzler J., Kaniecki K., Popik P., Smith J., Hashimoto K., Sporn J. (2020). R-(-)-ketamine modifies behavioral effects of morphine predicting efficacy as a novel therapy for opioid use disorder. Pharmacol. Biochem. Behav..

[129] Shafique H., Witkin J., Smith J., Kaniecki K., Sporn J., Holuj M., Krawczyk M., Kuziak A., Popik P. (2021). Rapid tolerance to behavioral effects of ethanol in rats: Prevention by R-(-)-ketamine. Pharmacol. Biochem. Behav..

[130] Highland J.N., Zanos P., Riggs L.M., Georgiou P., Clark S.M., Morris P.J., Moaddel R., Thomas C.J., Zarate C.A., Pereira E.F.R. (2021). Hydroxynorketamines: Pharmacology and Potential Therapeutic Applications. Pharmacol. Rev..

