# Supplementary material for: Ketamine, Esketamine, and Arketamine: Their Mechanisms of Action and Applications in the Treatment of Depression and Alleviation of Depressive Symptoms
Source: Biomedicines. 2024 Oct 9;12(10):2283. doi: 10.3390/biomedicines12102283 (PMC11505277; doi:10.3390/biomedicines12102283)
Supplement: Supplementary file 1 [file biomedicines-12-02283-s001.zip › biomedicines-3235339-supplementary.pdf]

Supplementary file

Direct links leading to the relevant sources used for information extraction for the presented research article:

https://journals.sagepub.com/doi/pdf/10.1177/0310057X0203000201

https://pubmed.ncbi.nlm.nih.gov/28731926/

https://pubmed.ncbi.nlm.nih.gov/30111247/

https://pubmed.ncbi.nlm.nih.gov/32915563/

https://pubmed.ncbi.nlm.nih.gov/37572245/

https://pubmed.ncbi.nlm.nih.gov/38185966/

https://pubmed.ncbi.nlm.nih.gov/31123547/

https://pubmed.ncbi.nlm.nih.gov/30702529/

https://pubmed.ncbi.nlm.nih.gov/38092601/

https://pubmed.ncbi.nlm.nih.gov/36991011/

https://pubmed.ncbi.nlm.nih.gov/38184443/

https://pubmed.ncbi.nlm.nih.gov/2650898/

https://pubmed.ncbi.nlm.nih.gov/32826629/

https://pubmed.ncbi.nlm.nih.gov/31348905/

https://pubmed.ncbi.nlm.nih.gov/35203296/

https://pubmed.ncbi.nlm.nih.gov/33487513/

https://pubmed.ncbi.nlm.nih.gov/32616209/

https://pubmed.ncbi.nlm.nih.gov/29945898/

https://link.springer.com/chapter/10.1007/978-3-030-79790-4_2

https://pubmed.ncbi.nlm.nih.gov/34269883/

https://pubmed.ncbi.nlm.nih.gov/32616207/

https://pubmed.ncbi.nlm.nih.gov/36527355/

https://pubmed.ncbi.nlm.nih.gov/9451486/

https://pubmed.ncbi.nlm.nih.gov/37245947/

https://pubmed.ncbi.nlm.nih.gov/33609274/

https://pubmed.ncbi.nlm.nih.gov/34052823/

https://pubmed.ncbi.nlm.nih.gov/34855448/

https://pubmed.ncbi.nlm.nih.gov/38697379/

https://pubmed.ncbi.nlm.nih.gov/37893038/

https://pubmed.ncbi.nlm.nih.gov/37149345/

https://pubmed.ncbi.nlm.nih.gov/30513009/

https://pubmed.ncbi.nlm.nih.gov/29322377/

https://pubmed.ncbi.nlm.nih.gov/38388704/

https://pubmed.ncbi.nlm.nih.gov/31519175/

https://pubmed.ncbi.nlm.nih.gov/35064103/

https://pubmed.ncbi.nlm.nih.gov/33617822/

https://pubmed.ncbi.nlm.nih.gov/31875248/

https://pubmed.ncbi.nlm.nih.gov/32224141/

https://pubmed.ncbi.nlm.nih.gov/32430328/

https://pubmed.ncbi.nlm.nih.gov/33155503/

https://pubmed.ncbi.nlm.nih.gov/35863316/

https://pubmed.ncbi.nlm.nih.gov/35182519/

https://pubmed.ncbi.nlm.nih.gov/33963284/

https://pubmed.ncbi.nlm.nih.gov/15718222/

https://pubmed.ncbi.nlm.nih.gov/26898322/

https://pubmed.ncbi.nlm.nih.gov/35779628/

https://pubmed.ncbi.nlm.nih.gov/35085752/

https://pubmed.ncbi.nlm.nih.gov/37593223/

https://pubmed.ncbi.nlm.nih.gov/36651238/

https://pubmed.ncbi.nlm.nih.gov/31579184/

https://pubmed.ncbi.nlm.nih.gov/28234212/

https://pubmed.ncbi.nlm.nih.gov/37488280/

https://pubmed.ncbi.nlm.nih.gov/25257213/

https://pubmed.ncbi.nlm.nih.gov/36793329/

https://pubmed.ncbi.nlm.nih.gov/29945718/

https://pubmed.ncbi.nlm.nih.gov/32252062/

https://pubmed.ncbi.nlm.nih.gov/32015715/

https://pubmed.ncbi.nlm.nih.gov/29282469/

https://pubmed.ncbi.nlm.nih.gov/31166571/

https://pubmed.ncbi.nlm.nih.gov/31109201/

https://pubmed.ncbi.nlm.nih.gov/31290965/

https://pubmed.ncbi.nlm.nih.gov/31734084/

https://pubmed.ncbi.nlm.nih.gov/16305457/

https://pubmed.ncbi.nlm.nih.gov/35948160/

https://pubmed.ncbi.nlm.nih.gov/30941749/

https://pubmed.ncbi.nlm.nih.gov/38666026/

https://pubmed.ncbi.nlm.nih.gov/32078034/

https://pubmed.ncbi.nlm.nih.gov/36871913/

https://pubmed.ncbi.nlm.nih.gov/37385001/

https://pubmed.ncbi.nlm.nih.gov/31415914/

https://pubmed.ncbi.nlm.nih.gov/34977960/

https://pubmed.ncbi.nlm.nih.gov/31135879/

https://pubmed.ncbi.nlm.nih.gov/31866390/

https://pubmed.ncbi.nlm.nih.gov/34313805/

https://pubmed.ncbi.nlm.nih.gov/36038019/

https://pubmed.ncbi.nlm.nih.gov/37083635/

https://pubmed.ncbi.nlm.nih.gov/38399409/

https://pubmed.ncbi.nlm.nih.gov/35234983/

https://pubmed.ncbi.nlm.nih.gov/35283178/

https://pubmed.ncbi.nlm.nih.gov/37477839/

https://pubmed.ncbi.nlm.nih.gov/36786865/

https://pubmed.ncbi.nlm.nih.gov/38928508/

https://pubmed.ncbi.nlm.nih.gov/21911285/

https://pubmed.ncbi.nlm.nih.gov/32563630/

https://pubmed.ncbi.nlm.nih.gov/33215721/

https://pubmed.ncbi.nlm.nih.gov/33403480/

https://pubmed.ncbi.nlm.nih.gov/34384829/

https://pubmed.ncbi.nlm.nih.gov/34688833/

https://pubmed.ncbi.nlm.nih.gov/9088882/

https://pubmed.ncbi.nlm.nih.gov/31514224/

https://pubmed.ncbi.nlm.nih.gov/25133650/

https://pubmed.ncbi.nlm.nih.gov/34819637/

https://pubmed.ncbi.nlm.nih.gov/30337374/

https://pubmed.ncbi.nlm.nih.gov/37793489/

https://pubmed.ncbi.nlm.nih.gov/33981335/

https://pubmed.ncbi.nlm.nih.gov/21892690/

https://pubmed.ncbi.nlm.nih.gov/36542981/

https://pubmed.ncbi.nlm.nih.gov/37146727/

https://pubmed.ncbi.nlm.nih.gov/32156500/

https://pubmed.ncbi.nlm.nih.gov/32592718/

https://pubmed.ncbi.nlm.nih.gov/32629972/

https://pubmed.ncbi.nlm.nih.gov/29532791/

https://pubmed.ncbi.nlm.nih.gov/30261975/

https://pubmed.ncbi.nlm.nih.gov/29174627/

https://pubmed.ncbi.nlm.nih.gov/28555075/

https://pubmed.ncbi.nlm.nih.gov/30032169/

https://pubmed.ncbi.nlm.nih.gov/30980851/

https://pubmed.ncbi.nlm.nih.gov/35594949/

https://pubmed.ncbi.nlm.nih.gov/36804535/

https://pubmed.ncbi.nlm.nih.gov/29307665/

https://pubmed.ncbi.nlm.nih.gov/33359641/

https://pubmed.ncbi.nlm.nih.gov/29111184/

https://pubmed.ncbi.nlm.nih.gov/31418048/

https://pubmed.ncbi.nlm.nih.gov/26327690/

https://pubmed.ncbi.nlm.nih.gov/32826629/

https://pubmed.ncbi.nlm.nih.gov/6989292/

https://pubmed.ncbi.nlm.nih.gov/3970799/

https://pubmed.ncbi.nlm.nih.gov/35188952/

https://pubmed.ncbi.nlm.nih.gov/8329260/

https://pubmed.ncbi.nlm.nih.gov/33896589/

https://pubmed.ncbi.nlm.nih.gov/29661378/

https://pubmed.ncbi.nlm.nih.gov/34316004/

https://pubmed.ncbi.nlm.nih.gov/28651788/

https://pubmed.ncbi.nlm.nih.gov/30140240/

https://pubmed.ncbi.nlm.nih.gov/32333922/

https://pubmed.ncbi.nlm.nih.gov/33577868/

https://pubmed.ncbi.nlm.nih.gov/33674359/
